# Supplementary figures and images for: Right Ventricular Strain by Magnetic Resonance Feature Tracking Is Largely Afterload-Dependent and Does Not Reflect Contractility: Validation by Combined Volumetry and Invasive Pressure Tracings
Source: Diagnostics (Basel). 2022 Dec 16;12(12):3183. doi: 10.3390/diagnostics12123183 (PMC9777736; doi:10.3390/diagnostics12123183)

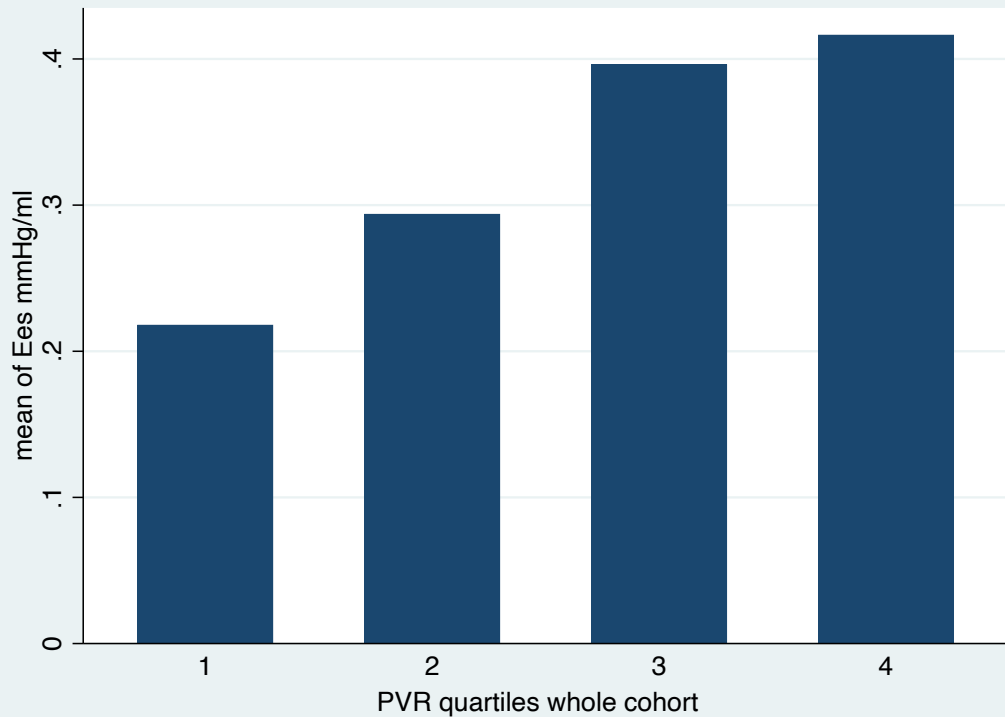

Supplement: Supplementary file 1 [file diagnostics-12-03183-s001.zip › Supplementary Figure S1 Ees over PVR quartiles whole cohort.pdf]

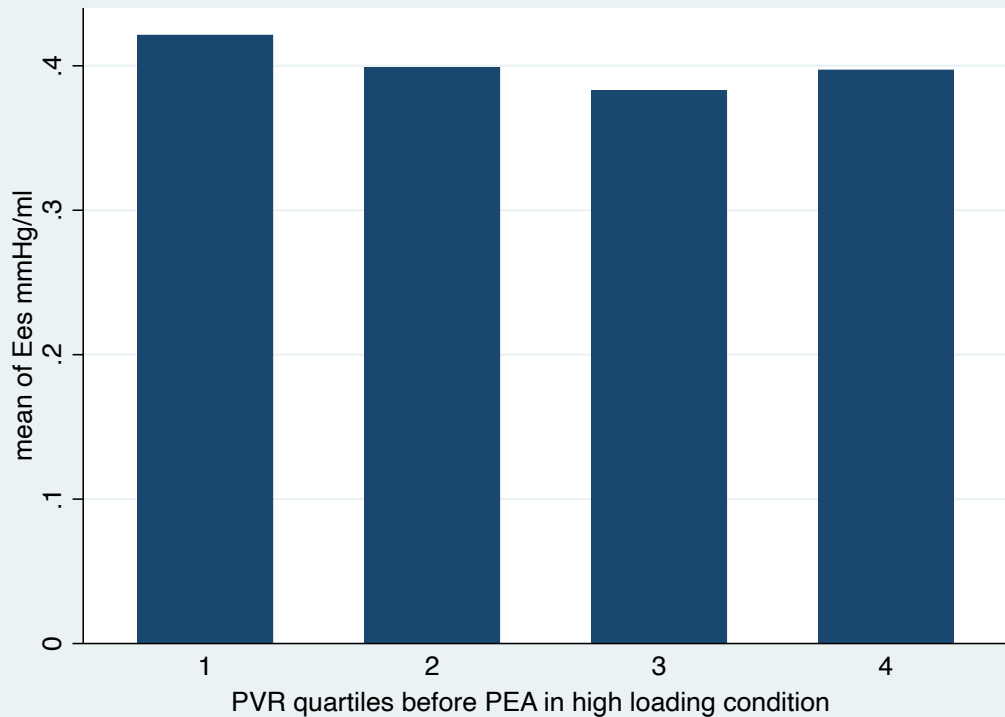

Supplement: Supplementary file 1 [file diagnostics-12-03183-s001.zip › Supplementary Figure S2 Ees over PVR quartiles high load.pdf]

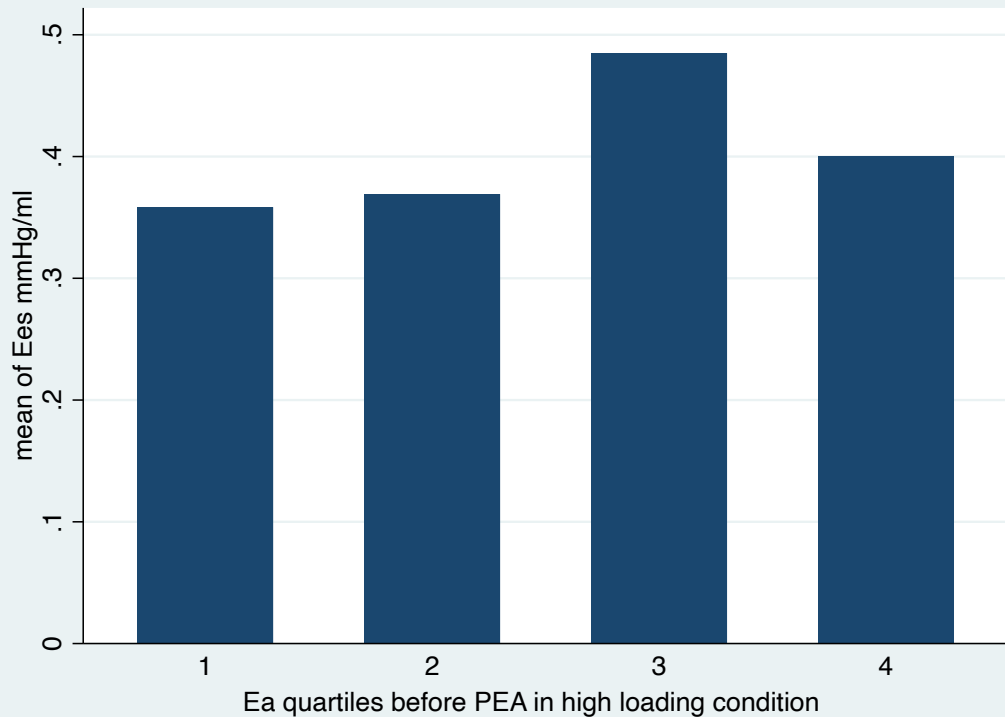

Supplement: Supplementary file 1 [file diagnostics-12-03183-s001.zip › Supplementary Figure S3 Ees over Ea quartiles high load.pdf]

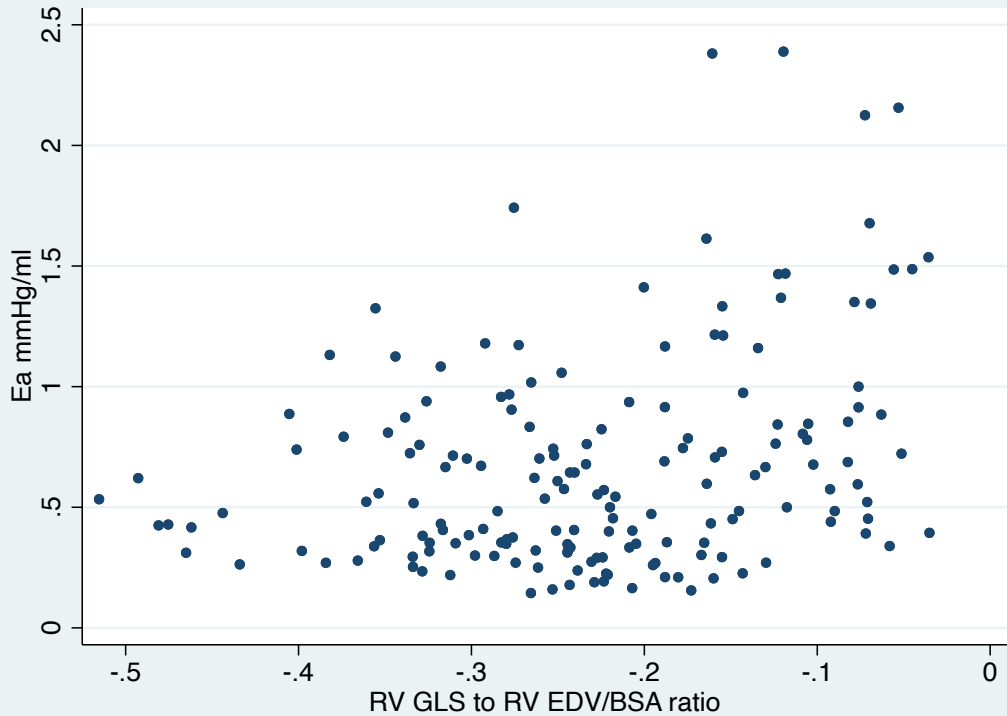

Supplement: Supplementary file 1 [file diagnostics-12-03183-s001.zip › Supplementary Figure S4 scatter GLS EDVi Ratio vs Ea.pdf]
